# Supplementary material for: Flexible auditory training, psychophysics, and enrichment of common marmosets with an automated, touchscreen-based system
Source: Nat Commun. 2022 Mar 28;13:1648. doi: 10.1038/s41467-022-29185-9 (PMC8960775; doi:10.1038/s41467-022-29185-9)
Supplement: Supplementary file 1 — Supplementary Information [file 41467_2022_29185_MOESM1_ESM.pdf]

Title:

Flexible auditory training, psychophysics, and enrichment of common marmosets with an automated, touchscreen-based system

Author list:

Calapai, A.*[1,2,3,4], Cabrera-Moreno, J.*[2,3,5,6], Moser, T.[3,5,6,7,8], Jeschke, M.[$ 2,3,4,5]

Affiliations:

1 Cognitive Neuroscience Laboratory, German Primate Center - Leibniz-Institute for Primate Research, Göttingen, Germany.

2 Cognitive Hearing in Primates (CHiP) Group, Auditory Neuroscience and Optogenetics Laboratory, German Primate Center - Leibniz-Institute for Primate Research, Göttingen, Germany.

3 Auditory Neuroscience and Optogenetics Laboratory, German Primate Center - Leibniz-Institute for Primate Research, Göttingen, Germany.

4 Leibniz ScienceCampus "Primate Cognition", Göttingen Germany

5 Institute for Auditory Neuroscience and InnerEarLab, University Medical Center Göttingen, 37075 Göttingen, Germany

6 Göttingen Graduate School for Neurosciences, Biophysics and Molecular Biosciences, University of Göttingen, 37075 Göttingen, Germany

7 Auditory Neuroscience Group and Synaptic Nanophysiology Group, Max Planck Institute for Multidisciplinary Sciences, 37077 Göttingen, Germany

8 Cluster of Excellence "Multiscale Bioimaging: from Molecular Machines to Networks of Excitable Cells" (MBExC), University of Göttingen, 37075 Göttingen

ORCIDs:

Calapai, A:	0000-0002-9098-7245
Cabrera-Moreno, J:	0000-0002-8762-1744
Moser, T:	0000-0001-7145-0533
Jeschke, M:	0000-0002-9109-8765

* These authors contributed equally.

[$] To whom correspondence should be addressed: mjeschke@dpz.eu

Supplementary materials and extended information

1. AUT versions performed by each animal
   Table S1

2. Overview of all automated unsupervised training protocols (AUT);
   TableS2

3. Acoustic Artificial Discrimination
   Figure S1
   Figure S2
   Table S3

4. Inter-Trial-Intervals analysis
   Figure S3
   Table S4

5. Additional device, training and trial timing information
   Figure S4

6. Example Videos:

   - Supplementary Movie 1: Habituation Phase. An animal can be seen exploring the behavioral chamber for the first time, file *Supplementary Movie 1.mp4*

   - Supplementary Movie 2: Mouthpiece-reward association. An animal can be seen approaching the mouthpiece and consuming the fluid reward. After the habituation phase, an experimenter remotely delivered fluid reward through the mouthpiece, file *Supplementary Movie 2.mp4*

   - Supplementary Movie 3.1: Touch-to-drink phase. An animal inside the mesh can be seen collecting pieces of marshmallows attached to the screen. Accidental touches of the screen during this process resulted in fluid reward delivered through the mouthpiece, file *Supplementary Movie 3_1.mp4*

   - Supplementary Movie 3.2: Touch-to-drink phase. The same animal of Video 3.1 touches the screen and consumes fluid reward from the mouthpiece, after all marshmallows have been collected, file *Supplementary Movie 3_2.mp4*

   - Supplementary Movie 4: An animal solves several trials on the final step of the Natural Discrimination task, file *Supplementary Movie 4.mp4*

1. AUT versions performed by each animal

Animal	AUT versions	Order of tasks
a	1, 2, 4, 5, 6, 8, 9,10	Pilot experiments, Natural Discrimination, Artificial Discrimination
b	1, 2, 4, 5, 6, 8, 9,10	Pilot experiments, Natural Discrimination, Artificial Discrimination
c	8, 9, 10	Natural Discrimination
d	9, 10	Natural Discrimination
e	9, 10	Natural Discrimination, Artificial Discrimination
f	8, 9, 10	Natural Discrimination
g	4, 5, 6, 8, 10	Pilot experiments, Artificial Discrimination, Natural Discrimination
h	4, 5, 6, 8, 10	Pilot experiments, Artificial Discrimination, Natural Discrimination
i	8, 9, 10	Natural Discrimination
j	9,10	Natural Discrimination
k	9, 10	Natural Discrimination
l	4	Pilot experiments
m	9, 10	Natural Discrimination
n	9, 10	Natural Discrimination

Table S1 – List of AUT versions performed by each animal. Data with versions 3 and 7 are not included in this manuscript due to technical issues with the RFID and to the nature of the experiment (control experiment testing pure visual assessment), respectively.

2. Overview of the AUT versions

AUT version	Description	Visual stimuli	Acoustic stimuli	Changes	notes
1	Protocol made of 40 steps composed of two main sections. The first focusing on training touch precision with a start-stimulus placed in the center of the screen that decreases in size until it reaches the final size of 3x3 cm. The second requiring a total of two interactions to obtain reward, one towards the start stimulus, placed in the center of the screen, and a second one towards either of the two visual stimuli, placed either left or right of the screen. Throughout this second section the distractor stimulus increases in size until it reaches the same size of the target stimulus.	Start-stimulus: White circle without background. Visual stimuli: two triangles, red and blue at opposite orientations, without background	Constant sine wave vs. no sound, matching the red and blue triangles respectively		
2	Same as version 1	Same as version 1	Same as version 1	A white background to the visual stimuli is added.	
3	Protocol made of 47 steps, composed of two main sections. The first section is the same as version 1. The second section is extended in the number of total steps, for finer distractor size increase.	Same as version 1	Same as version 1	17 new steps are added in the second section.	Data not processable due to tecnhical issues with the RFID
4	Protocol made of 54 steps, composed of three main sections. Section 1 is the same as version 1. The second section focusses on training the animal to reach for the target stimulus at different positions on the screen. From trial to trial the target is shown at variable eccentricities. The eccentricity is increased gradually until the edge of the screen is reached. The third section is the same as section 2 in version 3.	Start-stimulus: same as version 1. Visual stimuli: red cross and blue triangle embedded in a white background.	Simple train tone pulse vs. no sound, matching red cross and blue triangle respectively	The position of the visual stimuli is randomly assigned to right and left of the screen center, on a trial by trial basis. The identity of the visual stimuli has changed.	
5	Protocol made of 44 steps, composed of three main sections. The first section is the same as version 4 but with a reduced number of steps. In the second section, the acoustic stimulus, in each trial, is played from the left or right speaker, coherently with the side of the screen in which the visual stimulus was shown. The third section is the same as section 2 in version 3	Same as version 4	Same as version 4	Tthe visual and the acoustic stimuli are coherent in their source location. Decrease in number of steps in the first section.	
6	Protocols made of 44 steps, composed of three main sections. The first section one is the same as version 5. The second section is the same as version 5, but with the termination of the trial in case of no interactions after 7 seconds from stimuli onset (ignored trials). The visual and acoustic stimuli are presented at the same time.	Start-stimulus: White circle embedded in a blue background. Visual stimuli: same as version 4	Same as version 4	Implementation of ignored trials, with visual and acoustic stimuli disappearing after 7 seconds, and a new trial starts. Overlapping of visual and acoustic stimuli during presentation enhance coherence.	
7	Protocol made of 35 steps, comprised of three main sections. All sections are the same as version 6, but section contains less steps.	Start-stimulus: same as version 6. Visual stimuli: gray cross and gray triangle embedded in a gray background.	No sound	Alternative task for assessing visual discrimination. No sound / visual target association	Data now shown. Control experiment to assess pure visual discrimination
8	Protocol made of 44 steps, composed of three main sections. The first section is the same as version 6. In the second section the trial start button is shown at variable eccentricities. The eccentricity is increased gradually until the edge of the screen is reached. The third section is the same as version 6, but the feedback to the animal is enriched. The compound stimulus (acoustic and visual) is presented during the reward. The distractor and the target are shown in isolation after a wrong and correct response, respectively.	Same as version 6	Same as version 6	Overlapping of visual and acoustic stimuli together with reward delivery when a correct response is registered, the distractor stimulus is removed from screen. When wrong response is registered, the distractor stimulus remains and target stimulus is removed from the screen. In the second section the visual stimuli are replaced by the start stimulus.	
9	Same as version 8	Start-stimulus: same a version 6. Visual stimuli: face of baby marmoset vs. gray scale triangles within a gray square	Baby marmoset vocalization vs. train tone pulse, matching the baby face and the gray triangles composite respectively.	Change of visual and acoustic stimuli.	
10	Protocol made of 50 steps, comprised of three main sections. Same as version 8 but with added steps in the second section. For few animals, as a control condition, a different set of compound stimuli (acoustic and visual) are used – see Artificial Discrimination task in the methods sections. This is the final and most successfully protocol, described in the method section of the original manuscript.	Start-stimulus: same as version 6. Visual stimuli: same as version 9. Visual stimuli for the control condition referred as Artificial Discrimination: RGB geometric figure embedded in a yellow background vs. gray scale triangles within a gray square.	Acoustic stimuli: same as version 9. Acoustic stimuli for the control condition referred as Artificial Discrimination: simple train used as acoustic stimulus in version 9 vs. two tones train pulse (complex train), (matching the RGB geometric figure and the gray triangles composite respectively)	A new set of steps are added in the second section. A control condition is implemented for few animals	

Table S2; overview of all versions of the automated unsupervised training protocol (AUT)

3. Artificial Discrimination

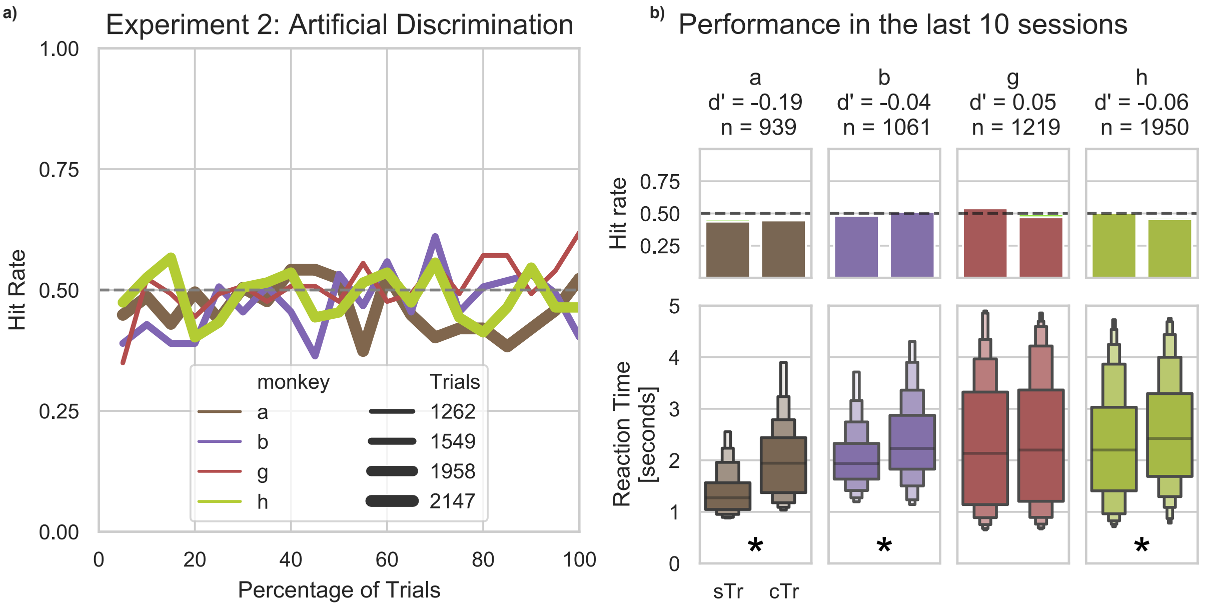

Figure S1. A; Hit rate as a function of percentage of trials performed in the Artificial Discrimination. Line thickness represents the total number of trials of each animal in this task. Dashed line at 0.5 represents chance level. Hit rate for each animal is represented as a function of the animal's percentage of trials performed and is grouped into bins of 5% of trials. B; hit rate as a function of stimulus type ("sTr" for the train of pure tones, "cTr" for the train of alternating pure tones) in the last 10 sessions, with corresponding number of trials and sensitivity index (d') above each bar plot. Letter-value plots of the reaction times were plotted for each stimulus type separately. The central box defines the median and 25th up to 75th percentiles. Successively narrower boxes are drawn between the 1/8th and 7/8th, the 1/16th and 15/16th, and so on percentile. Stars in reaction time panels represent significant difference in reaction times between the two stimuli at a Bonferroni post-hoc corrected Kruskal-Wallis Test (one-sided test), for a detail statistics summary see Table S3.

This variant of the audio-visual association experiment (Figure S1) employed two artificially constructed stimuli consisting of trains of Pure Tones: a simple Train (sTr) composed of a repeating pure tone in the range between 1.5 and 3.5 kHz (repetition rate of 3.3 Hz, one frequency chosen per animal); and a complex Train (cTr), consisting of a repeating pattern of 2 pure tones alternating between a fundamental frequency (chosen from 1.5 to 3.5 kHz, one fundamental frequency per animal) and a frequency 42 % higher (which was always larger than the minimum discriminable frequency difference [1]). The sTr was associated with a composite of three grey scaled triangles embedded in a 3x3cm grey square, and the complex train with a colored geometric figure embedded in a 3x3cm yellow square. None of the tested animals performed above chance although differences in reaction times were observed. Note that one

marmoset (animal e), that performed on this task was not included into Figure S1 and table S3 due to technical problems during the sessions.

Animals	Stimulus	Hitrate	Trials	d'	Binomial test on performance (Figure S1)			Kruskal-Wallis test on Reaction Times (Figure S1)					
					N (w/o ignored)	Degrees of freedom	Binomial Test (adjusted p-value)	Median	IQR	N	Degrees of freedom	Test Statistics	Kruskal-Wallis (adjusted p-value)
a	sTr	0.45	460	-0.19	456	1	1	1.27	0.52	223	1	89.848	**2.06E-20**
	cTr	0.44	497		489	1	1	1.94	1.06	207	1		
b	sTr	0.51	385	-0.04	380	1	1	1.94	0.69	215	1	21.188	**3.33E-05**
	cTr	0.48	405		404	1	1	2.23	1.04	215	1		
g	sTr	0.47	651	0.05	636	1	1	2.14	2.18	287	1	0.049	**1**
	cTr	0.54	591		585	1	0.15	2.20	2.16	260	1		
h	sTr	0.46	1017	-0.06	1004	1	1	2.20	1.62	457	1	12.141	**0.003**
	cTr	0.50	963		954	1	1	2.42	1.61	419	1		

Table S3, Summary statistics for the variant "acoustic discrimination" of the audio-visual association experiment across animal and stimuli (Figure S1). Significant values are indicated in bold font. D-prime value is provided as indication of the sensitivity of each animal on given task. Columns under "Kruskal-Wallis test on Reaction Times (Figure S1)" report information regarding the statistical difference of the reaction time to the sTr and the cTr stimuli, with p-values adjusted with a post-hoc Bonferroni correction for multiple comparisons. Columns “Binomial test on performance (Figure S1)” report information regarding the statistical deviations of performance (across stimuli and task type) from a theoretically expected distribution of observations (one-sided), with p-values adjusted with a post-hoc Bonferroni correction for multiple comparisons.

With the aim of testing further attempts on how to train animals to perform artificial discrimination, we developed an alternative approach (AD_2, Fig. S2) which was designed as a continuation of the acoustic discrimination AUT described in the main text. The idea behind AD_2 was to introduce a previously unknown discrimination by contrasting a new stimulus with stimuli for which a stimulus-response association already exists and then successively reducing the percentage of trials with known contrast while increasing the percentage of trials for the unknown stimulus contrast.

In other words: initially animals know to discriminate the simple train (sTr) from the vocalization (voc), by touching a geometric figure or a marmoset face, respectively. The final goal is to discriminate a simple train from a complex train (cTr) by choosing appropriate geometric patterns (triangles vs. keyhole; for a stimulus description and correct visual response see Fig. S2A). At the beginning of the AD_2, the 2 alternative stimuli available at every trial are the already acquired ones, namely the sTr or the voc. Throughout the steps of the procedure, the voc is replaced, in increasing proportion of trials, by the cTr. Therefore, while the sTr always had 50% chance of being a target, the voc probability decreased throughout the procedure, in favour of cTr, the probability of which increased in steps of 4 % per level. The resulting 12 possible trial types can be seen in Fig. S2A. Moreover, to move between steps of the AD_2 we modified the performance evaluation algorithm such that increases in step occurred after 80 % of trials or more were correct within a window of 24 trials and step-downs already

occurred if 45 % of trials or less were correct within a 24-trial window. The AD_2 starts from step 50 (final step of the acoustic discrimination AUT) and gradually increases the percentage of trial types 9, 10, 11 and 12 while decreasing the percentage of trial types with an already trained stimulus-response association (1, 2, 3 and 4). In all steps the animal could correctly perform each trial by selecting a known stimulus-response (trial types 5, 6) or excluding a known response (trial types 7, 8). On each of the 12 steps of the AD_2 the proportion of new trial types (9, 10, 11, 12) increased by 2.1 % per step while trial types with known stimulus-response association (1, 2, 3, 4) decreased over 6 steps by 4.2 % per step. To assist in learning the new stimulus-response association between the cTr and the keyhole pattern, trial types with a vocalization (voc; trial types 5, 6) or the cTr paired with a marmoset's face as distractor (7, 8) were first introduced and increased in likelihood along the stair case until step 56 (2.1 % per trial type and step) after which they were successively eliminated until step 62 (2.1 % per trial type and step). Animal a quickly progressed through the AD_2 reaching the final step (62) for the first time in session 3 (after 1940 trials from the start of the AD_2 procedure, Fig. S2B, C) and stabilized on step 62 from session 9 (after 4222 trials) when the animal quickly recovered from previous step-downs in sessions 5 to 8 (Fig. S2B, C). To assess whether animal a had acquired the final discrimination after stabilizing, we compared only trials where the sTr had to be discriminated from the cTr with their respective visual targets (trial types 9, 10, 11 and 12). Throughout 3552 total trials animal a chose the keyhole visual target after cTr significantly more often than after sTr presentation (Fisher's Exact test, $p = 1.6*10^{-71}$; cTr hit rate = 62 %, sTr hit rate = 68 %, see Fig. S2D).

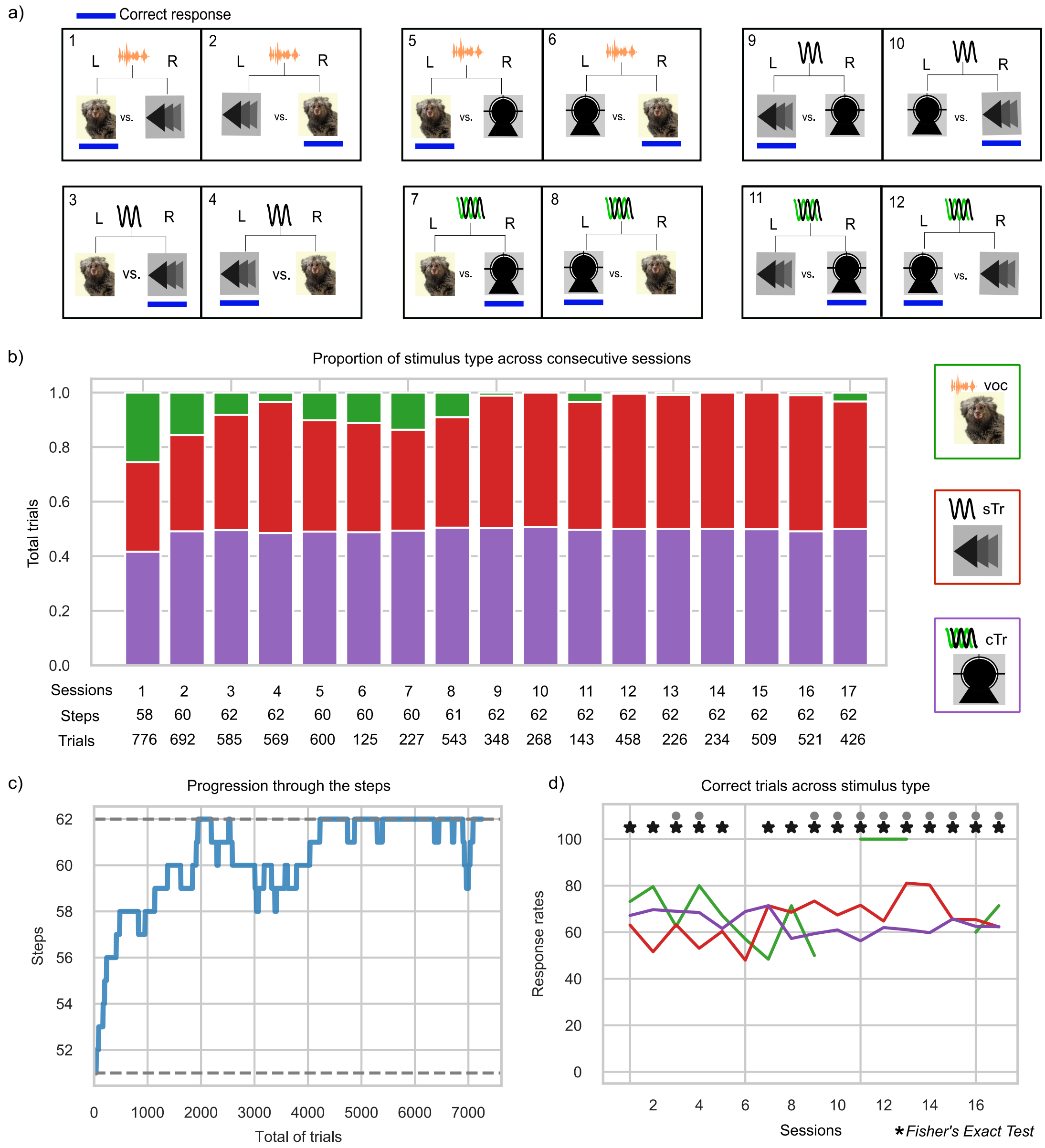

Figure S2. Performance of animal a on version 2 of the Artificial Discrimination task.

A) Graphical representation of the 12 trial types employed within the AUT, Blue bar under visual indicator shows correct response. B) Shows the percentage of every auditory target across consecutive sessions. Numbers at the bottom indicate the number, highest step reached in that session and the total number of trials for every session. Colors of the panels on the right corresponds to the three targets. C) Learning curve of animal a along the different steps of the task. D) Depicts the hit rates for the three individual targets (sTr, Voc and cTr) across consecutive sessions. The grey dot on top marks, for every session, whether the highest step (step 62) in the AUT was reached. The star represents a significant difference between hit-rate and error-rate between trial types 9, 10 vs 11, 12 or sTr and cTr (Fisher's Exact Test, two sided), p-values listed by session order (3.11e-13, 2.14e-07, 4.24e-14, 1.92e-07, 3.08e-07, 8.05e-02, 2.31e-09, 8.97e-09, 7.26e-10, 3.22e-06, 1.08e-03, 5.41e-09, 8.60e-11, 4.16e-10, 1.74e-12, 3.09e-10, 6.90e-08).

4. Inter-Trial-Intervals analysis:

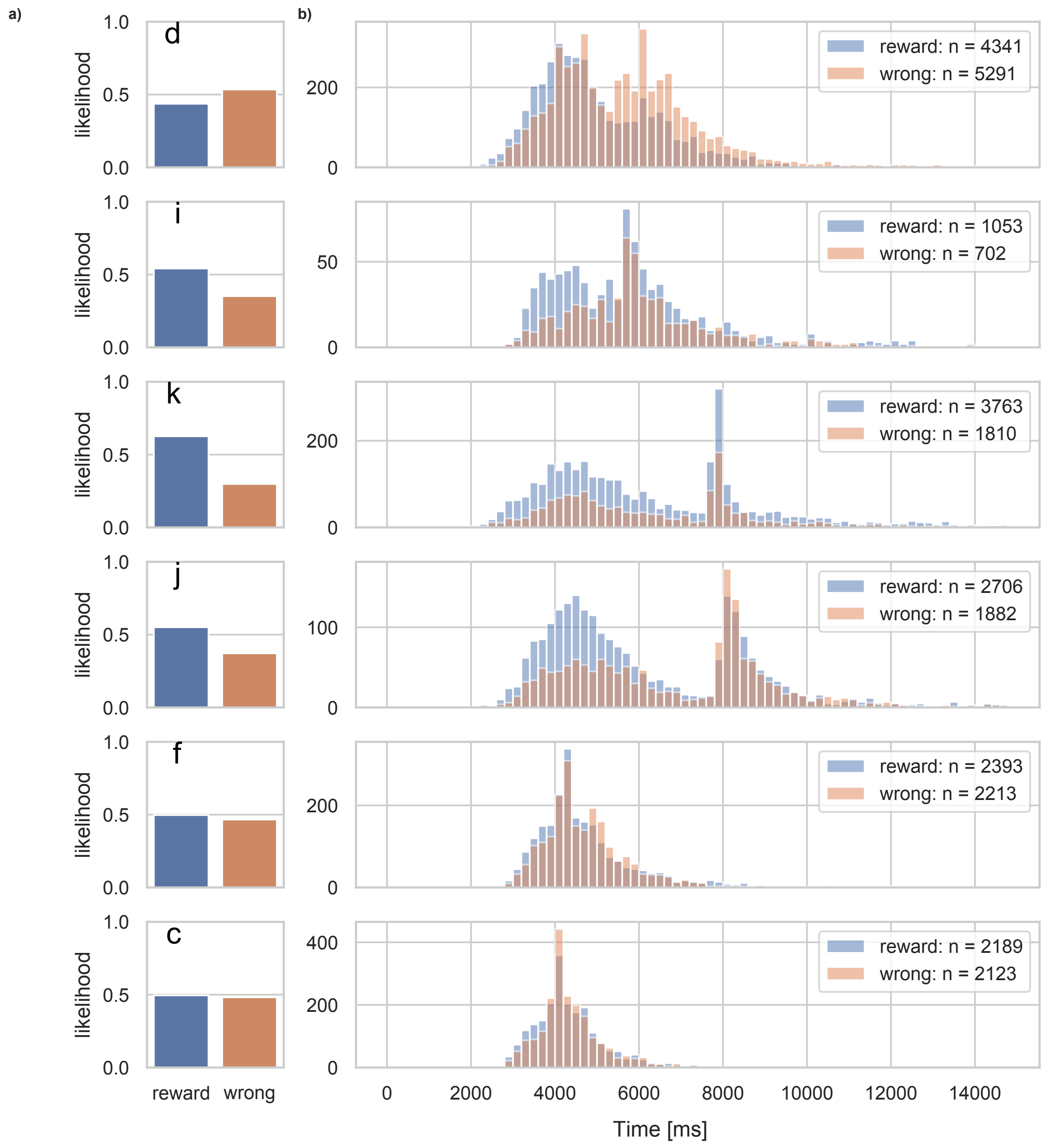

Figure S3 – Visualization of Inter-Trial-Intervals in the automated, unsupervised training (AUT - version 10). A; bar plots indicating, for each animal, the likelihood of a new trial being initiated within 30 seconds from a correct (blue) or a wrong (orange) previous trial. Letters in the bar plots indicate the animals. B; histograms reporting the distribution of inter-trial-interval of each of the 6 animals. Average and standard deviation are given in Table S4.

For animals that underwent the final AUT (version 10) and performed the acoustic discrimination task described in the main text, we quantified the likelihood of initiating a trial after a correct or a wrong response (within 30 seconds) and analysed the time (in seconds) between consecutive trials for correct and wrong trials separately (Figure

S3). Note that after a wrong response a timeout of 2.5 to 5 seconds was used, such that new trials could not be initiated and touches were ignored before the timeout ended. For correct responses instead, the trial was available after a time interval of 0.8 to 2.5 seconds. As a result, we observed differences in likelihood of initiating a new trial (Figure S3A) and in the distribution of inter-trial intervals (Figure S3B) after correct vs. wrong responses in animals who ultimately acquired the acoustic discrimination task (animals d, i, k, j).

Animal	Mean Correct	Mean Wrong	std Correct	std Wrong
c	4604.57	4626.39	3040.61	3010.72
d	5700.33	6474.76	4384.6	4748.27
f	5123.12	5210.68	3533.57	4020.49
i	7041.53	7798.84	6594.45	7522.4
j	7459.02	8142.14	6103.64	5879.97
k	7702.85	7748.64	6614.6	6070.68
total	6271.9	6666.91	5045.24	5208.75

Table S4 – Average inter-trial-intervals (in milliseconds) and standard deviation across animals in the AUT (version 10), for correct and wrong trials.

5. Additional device, training and trial timing information

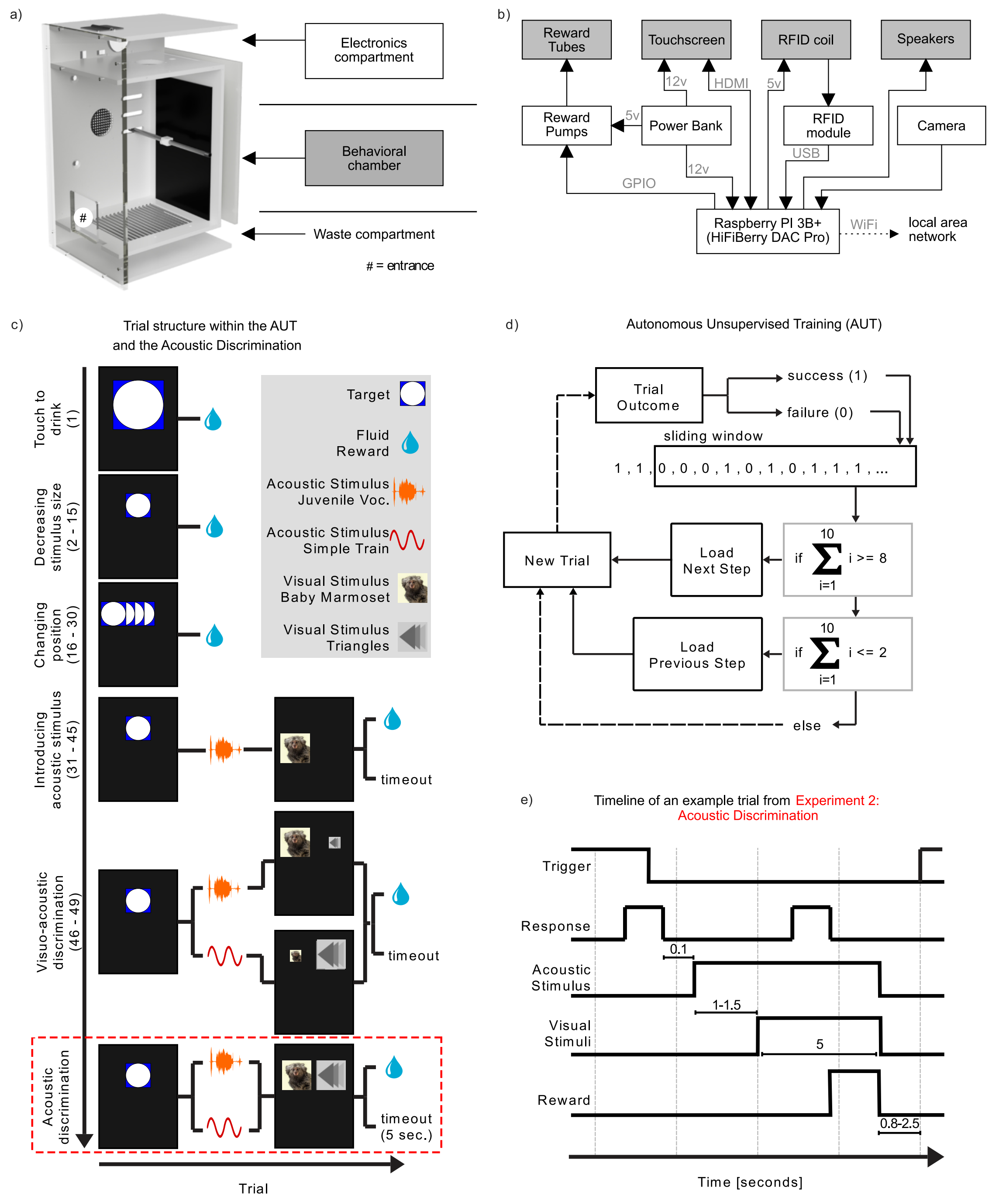

Figure S4 – A) rendering of the MXBI without a side panel to show the three internal compartments. B) schematics of the connections and flow of information between individual components of the behavioral chamber (grey background) and of the electronics compartment (white background). C) basic trial structure from the animals' perspective, across six exemplary steps of the automated protocol (AUT). D) schematic representation of the logic of the recursive algorithm responsible for monitoring the animal's trial-by-trial performance and changing the step accordingly. E) example trial timeline (including the animal's response) for a successful trial at the last step of the AUT protocol, the audio-visual association.

References

1. Osmanski, M. S., Song, X., Guo, Y. & Wang, X. Frequency discrimination in the common marmoset (Callithrix jacchus). *Hearing Research* **341**, 1–8 (2016).
