# Supplementary material for: Flexible auditory training, psychophysics, and enrichment of common marmosets with an automated, touchscreen-based system
Source: Nat Commun. 2022 Mar 28;13:1648. doi: 10.1038/s41467-022-29185-9 (PMC8960775; doi:10.1038/s41467-022-29185-9)
Supplement: Supplementary file 9 — Reporting Summary [file 41467_2022_29185_MOESM9_ESM.pdf]

nature portfolio

Corresponding author(s): Marcus Jeschke

Last updated by author(s): Feb 17, 2022

Reporting Summary

Nature Portfolio wishes to improve the reproducibility of the work that we publish. This form provides structure for consistency and transparency in reporting. For further information on Nature Portfolio policies, see our Editorial Policies and the Editorial Policy Checklist.

Statistics

For all statistical analyses, confirm that the following items are present in the figure legend, table legend, main text, or Methods section.

n/a	Confirmed	
☐	☒	The exact sample size (n) for each experimental group/condition, given as a discrete number and unit of measurement
☐	☒	A statement on whether measurements were taken from distinct samples or whether the same sample was measured repeatedly
☐	☒	The statistical test(s) used AND whether they are one- or two-sided<br>*Only common tests should be described solely by name; describe more complex techniques in the Methods section.*
☐	☒	A description of all covariates tested
☐	☒	A description of any assumptions or corrections, such as tests of normality and adjustment for multiple comparisons
☐	☒	A full description of the statistical parameters including central tendency (e.g. means) or other basic estimates (e.g. regression coefficient) AND variation (e.g. standard deviation) or associated estimates of uncertainty (e.g. confidence intervals)
☐	☒	For null hypothesis testing, the test statistic (e.g. F, t, r) with confidence intervals, effect sizes, degrees of freedom and P value noted<br>*Give P values as exact values whenever suitable.*
☒	☐	For Bayesian analysis, information on the choice of priors and Markov chain Monte Carlo settings
☒	☐	For hierarchical and complex designs, identification of the appropriate level for tests and full reporting of outcomes
☒	☐	Estimates of effect sizes (e.g. Cohen's d, Pearson's r), indicating how they were calculated

Our web collection on statistics for biologists contains articles on many of the points above.

Software and code

Policy information about availability of computer code

Data collection	Python 3.5.3 with the following modules: tkinter 8.6, numpy 1.12.1, RPi.GPIO 0.6.5, pyaudio 0.2.11
Data analysis	Python 3.9 with the following modules installed: seaborn 0.11.1, pingouin 0.3.10 and psignifit 0.1. The code is available at a dedicated Github repository (https://github.com/CHiP-Lab/mXBI) and Zenodo (https://doi.org/10.5281/zenodo.6139297).

For manuscripts utilizing custom algorithms or software that are central to the research but not yet described in published literature, software must be made available to editors and reviewers. We strongly encourage code deposition in a community repository (e.g. GitHub). See the Nature Portfolio guidelines for submitting code & software for further information.

Data

Policy information about availability of data

All manuscripts must include a data availability statement. This statement should provide the following information, where applicable:

- Accession codes, unique identifiers, or web links for publicly available datasets
- A description of any restrictions on data availability
- For clinical datasets or third party data, please ensure that the statement adheres to our policy

The data generated during and/or analyzed for the current study are available at GitHub (https://github.com/CHiP-Lab/mXBI) and Zenodo (https://doi.org/10.5281/zenodo.6139297).

Field-specific reporting

Please select the one below that is the best fit for your research. If you are not sure, read the appropriate sections before making your selection.

☐ Life sciences ☒ Behavioural & social sciences ☐ Ecological, evolutionary & environmental sciences

For a reference copy of the document with all sections, see nature.com/documents/nr-reporting-summary-flat.pdf

Behavioural & social sciences study design

All studies must disclose on these points even when the disclosure is negative.

Study description	Quantitative study measuring the level of engagement of marmoset monkeys with a cage-based device. Training efficacy and general cognitive abilities of the animals were quantified and compared across animals and training stra
Research sample	14 Marmoset monkeys (6 females, 8 males) between 24 and 84 months of age at the beginning of the experiments and living in the animal facility of the research institute. The study sample was made available for the study by the colony management of the German Primate Center and not chosen by the experimenters. We consider this sample to be representative.
Sampling strategy	No sampling strategy was adopted. All animals available to the research group were given access to the cage-based device.
Data collection	The cage-based device was controlled by a single-board computer (Raspberry Pi) which registered information of every trial of the cognitive task as well as every interaction of the animals with the touchscreen and the RFID reader. For any given session the computer automatically compiled a text based document with all these information and routinely uploaded this datafile to a centralized server. Multiple devices were running at the same time and therefore data collection from multiple groups of animal often happened in parallel. Occasionally, animal care takers entered colony rooms were the devices were active to perform animal husbandry tasks such as feeding etc. No researcher was present in the colony rooms during data collection. Researchers were not blinded to experimental condition and/or the study hypothesis.
Timing	Data collection started on the 27.11.2018 and ended on 16.07.2021. Breaks in between occurred exclusively to debug and refine the data collection procedure. Data collection mostly occurred from Monday to Friday with some sessions taking place on Saturday and Sunday. Normally 5 sessions per week. The sessions mostly happened between 11.00am to 5:00pm
Data exclusions	No data has been excluded
Non-participation	One animal that participated in the initial stages of the study did not continue because it was later on assigned to a different project
Randomization	At the last stage of the study, where different stimuli are used to train acoustic discrimination, two animals (housed together) were randomly assigned to run under a control condition in which the order of first exposure to specific combinations of stimuli was opposite with respect to the order the remainder of the animals

Reporting for specific materials, systems and methods

We require information from authors about some types of materials, experimental systems and methods used in many studies. Here, indicate whether each material, system or method listed is relevant to your study. If you are not sure if a list item applies to your research, read the appropriate section before selecting a response.

Materials & experimental systems

n/a	Involved in the study	
☒	☐	Antibodies
☒	☐	Eukaryotic cell lines
☒	☐	Palaeontology and archaeology
☐	☒	Animals and other organisms
☒	☐	Human research participants
☒	☐	Clinical data
☒	☐	Dual use research of concern

Methods

n/a	Involved in the study	
☒	☐	ChIP-seq
☒	☐	Flow cytometry
☒	☐	MRI-based neuroimaging

Animals and other organisms

Policy information about studies involving animals; ARRIVE guidelines recommended for reporting animal research

Laboratory animals	14 Marmoset monkeys (6 females, 8 males) between 24 and 84 months of age at the beginning of the experiments.
Wild animals	No wild animals were used in this study.

Field-collected samples	No field collected samples were used in this study.
Ethics oversight	Approved by the responsible regional government office (Niedersaechsisches Landesamt fuer Verbraucherschutz und Lebensmittelsicherheit - LAVES, Permit No. 18/2976) , as well as the ethics committee of the German Primate Center (Permit No. E1-20_4_18).

Note that full information on the approval of the study protocol must also be provided in the manuscript.
